# A high throughput cell stretch device for investigating mechanobiology *in vitro*

**DOI:** 10.1063/5.0206852

**Published:** 2024-06-26

**Authors:** Stephen J. P. Pratt, Christopher M. Plunkett, Guray Kuzu, Ton Trinh, Joshua Barbara, Paula Choconta, Doug Quackenbush, Truc Huynh, Anders Smith, S. Whitney Barnes, Joel New, James Pierce, John R. Walker, James Mainquist, Frederick J. King, Jimmy Elliott, Scott Hammack, Rebekah S. Decker

**Affiliations:** Novartis, Biomedical Research 10675 John Jay Hopkins Dr, San Diego, California 92121, USA

## Abstract

Mechanobiology is a rapidly advancing field, with growing evidence that mechanical signaling plays key roles in health and disease. To accelerate mechanobiology-based drug discovery, novel *in vitro* systems are needed that enable mechanical perturbation of cells in a format amenable to high throughput screening. Here, both a mechanical stretch device and 192-well silicone flexible linear stretch plate were designed and fabricated to meet high throughput technology needs for cell stretch-based applications. To demonstrate the utility of the stretch plate in automation and screening, cell dispensing, liquid handling, high content imaging, and high throughput sequencing platforms were employed. Using this system, an assay was developed as a biological validation and proof-of-concept readout for screening. A mechano-transcriptional stretch response was characterized using focused gene expression profiling measured by RNA-mediated oligonucleotide Annealing, Selection, and Ligation with Next-Gen sequencing. Using articular chondrocytes, a gene expression signature containing stretch responsive genes relevant to cartilage homeostasis and disease was identified. The possibility for integration of other stretch sensitive cell types (e.g., cardiovascular, airway, bladder, gut, and musculoskeletal), in combination with alternative phenotypic readouts (e.g., protein expression, proliferation, or spatial alignment), broadens the scope of high throughput stretch and allows for wider adoption by the research community. This high throughput mechanical stress device fills an unmet need in phenotypic screening technology to support drug discovery in mechanobiology-based disease areas.

## INTRODUCTION

Cells sense and respond to mechanical forces derived from cell-to-cell interactions, cell-to-ECM interactions, and macro movements within the body. A cell's response or adaptation depends on its ability to distinguish physical cues such as tension, compression, shear stress, hydraulic pressure, and stiffness. For example, tensile stresses in the plasma membrane mechanically gate the ion channels Piezo1 and Piezo2,^1–3^ nuclear compression activates entry of the transcriptional regulator Yes-associated protein (YAP),^4,5^ GPR68 senses extracellular fluid flow shear stress,^6^ hydraulic pressure activates TRPM7,^7^ and integrin-activated cytoskeletal contractility senses substrate stiffness.^8–14^ Once applied, forces are translated via sensor-specific mechanotransduction pathways to initiate some of the most fundamental cell functions including transcription,^15^ growth,^16^ division,^17^ polarity,^18^ differentiation,^19^ and migration.^7,20–22^ However, the absence of mechanosensation in cells can be as disruptive as whole organ dysfunction.^23–25^ In addition, adaption mechanisms to mechanical stress may be employed by cells such as stress buffering,^26–28^ persistent mechanical memory,^29–34^ or reciprocal modification of the extracellular environment.^35–42^ Yet, pathological adaptation and disease can ensue when mechanical homeostasis has gone awry,^43^ including atherosclerosis,^44–47^ osteoarthritis,^48–52^ tendinopathy,^53–56^ or cancer.^12,57–59^

This necessity of cellular mechanosensation together with the growing evidence for abnormal mechanical signaling in disease pathology, illuminates unique avenues for mechanobiology-based drug discovery and therapeutic intervention. The development of mechanobiology research tools that can reliably apply specific or combinatorial mechanical stress types, and can be scaled up in throughput, will further facilitate screening-based drug development, new drug target identification, and target validation. However, one challenge is a lack of devices that apply mechanical stress in high throughput and integrate into screening and automation systems. One such mechanical stress type relevant to a variety of organs is stretch.^24,25,60–68^ To meet this technology need, a custom *in vitro* cell stretch device and stretchable silicone 192-well plate were designed and fabricated. The principal biological aim of this *in vitro* stretch device is to apply tensile strains and phenocopy cell responses that occur *in vivo*. Previous work using chondrocytes supports this objective, whereby many of the anabolic and catabolic responses that occur with physiologic 3D compression of cartilage^69–79^ can also be replicated with *in vitro* cell stretch.^80–90^ Therefore, chondrocytes were utilized as a representative mechanoresponsive cell type to demonstrate proof of concept cellular responses to stretch and utility of the stretch device. To this end, the current study describes differential gene expression with stretch, gene set enrichment analysis, and a curated signature of stretch responsive genes. Full plate transcriptional analysis per well demonstrates applicability of the gene signature readout for screening. Finally, stretch plate integration with automated cell dispensing and high content imaging systems demonstrates high throughput and screening compatibility.

## RESULTS

**FIG. 1.**
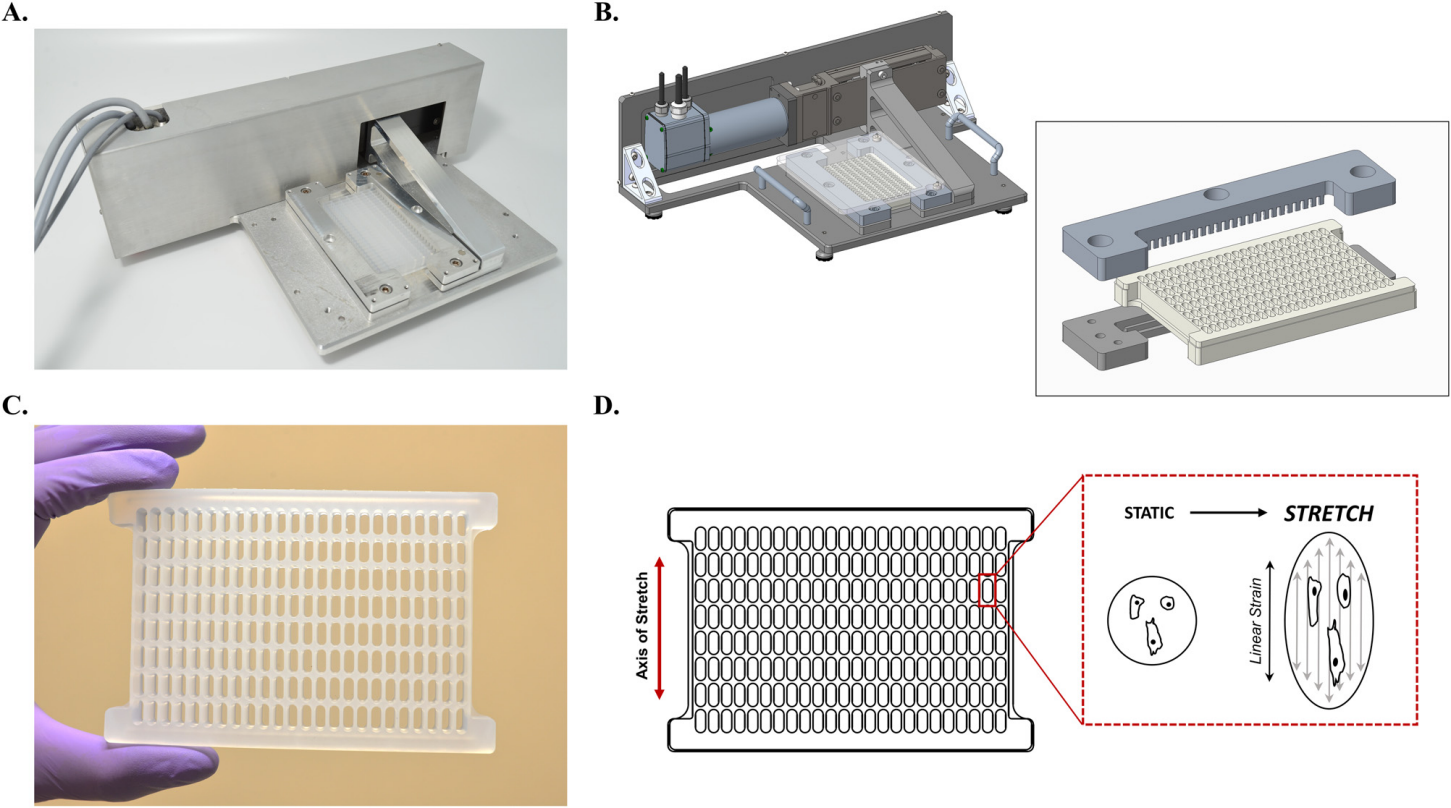
High throughput stretch device and 192-well plate. (a) The incubator compatible stretch device hardware, with 192-well linear stretch plate secured in the clamp, is shown. The device is operated using a LabVIEW user interface. (b) Computer-aided design (CAD) image of the stretch device shows a hardware configuration that includes a linear drive with one fixed and one moving plate clamp. Exploded view CAD image exhibits clamps designed to grip the first and last row of each plate using a series of teeth that partially inset into each well and an interlocking feature on the plate's underside. (c) Stretchable 192-well assay plates were injection molded using silicone elastomer resin and collagen coated for cell attachment. Individual wells have 250 *μ*l volume capacity, 16.92 mm^2^ well surface area, and high optical clarity well bottom for imaging. (d) Stretch is applied to the plate and attached cells in a linear direction along the plate column axis.

A custom stretch hardware device and accompanying silicone linear stretch plate were developed (Fig. 1, supplementary material dataset 1). The hardware device is incubator compatible and consists of a linear drive with one fixed and one moving plate clamp for application of stretch to the plate along a single axis [Figs. 1(a) and 1(b), supplementary material Video 1). To achieve a robust and even hold across the top and bottom plate edges, plate clamps were constructed to grip the first and last row of each plate using a series of teeth that partially insert into each well and an interlocking feature on the plate's underside [Fig. 1(b), inset]. Stretch plates were fabricated using injection molded NUSIL MED-4930 silicone elastomer resin in a custom steel mold. Plates were designed in 192-well format (8 rows × 24 columns) with extensions along the row axis at the top and bottom of the plate for additional material to clamp; however, the external contour fits ANSI standard plate dimensions [Fig. 1(c)]. Individual wells have 250 *μ*l volume capacity, 16.92 mm^2^ well surface area, and a high optical clarity well bottom for imaging. Linear stretch is applied to the plate and attached cells along the column axis [Fig. 1(d)], with a 50% stretch capacity for the stretch plate (supplementary material Fig. 1). Customized LabVIEW-based software is used to control stretch parameters such as time, magnitude, cycle frequency (Hz), and waveform.

**FIG. 2.**
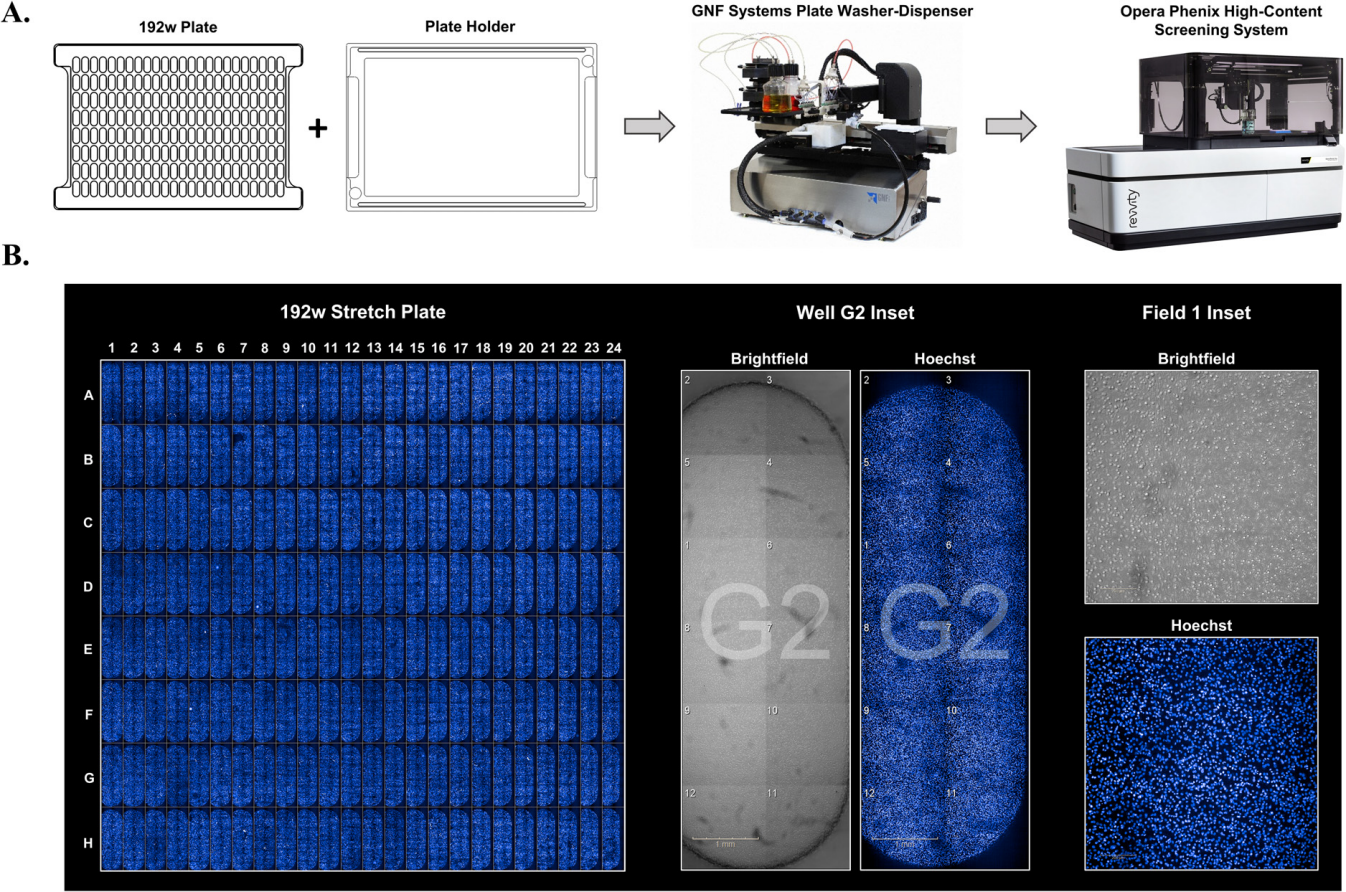
Stretch plate automation compatibility. (a) The workflow graphic shows the 192-well linear stretch plate with an aluminum plate holder that was constructed to support the stretch plate and allow automation gripping mechanisms to firmly clasp the plate. The combined plate and holder facilitate the use of automated platforms such as the GNF Systems Plate Washer-Dispenser for collagen coating and cell dispensing into stretch plates, and Opera Phenix High-Content Screening System to image cells. (b) Images show Hoechst-stained nuclei from human primary chondrocytes dispensed into collagen coated stretch plates and after an overnight attachment. The left image (192w stretch plate) is a full plate view composite of stitched and z-stack images from each of 192 wells. Middle panels (well G2 inset) show brightfield and fluorescent images from a representative stitched single well, and right panels (field 1 inset) show single 10× images from the stitched single well series for a higher magnification view of Hoechst-stained nuclei and brightfield imaging of chondrocytes. Images were reproduced with permission from GNF Systems and Revvity.

First, automation and biological compatibility of the plate were examined. *In vitro* cell stretch has been previously applied to chondrocytes to investigate mechanical activation of gene expression^80–90^ and were utilized here. Due to the flexibility of the silicone, an aluminum plate holder was constructed to support the stretch plate, prevent it from sagging in the z-direction, and allow automation gripping mechanisms to firmly clasp the plate (supplementary material Fig. 2). To demonstrate stretch plate and plate holder integration with automation [Fig. 2(a)], plates were first plasma treated and collagen coated for chondrocyte adhesion using an automated plate washer for liquid handling. Then, chondrocytes were plated using an automated cell dispenser and imaged using a high content imaging platform. Full plate view imaging of Hoechst-stained cultured cells shows consistent and repeatable cell seeding across the plate [Fig. 2(b)] and evenly dispersed confluent cell monolayers [Fig. 2(b), insets].

**FIG. 3.**
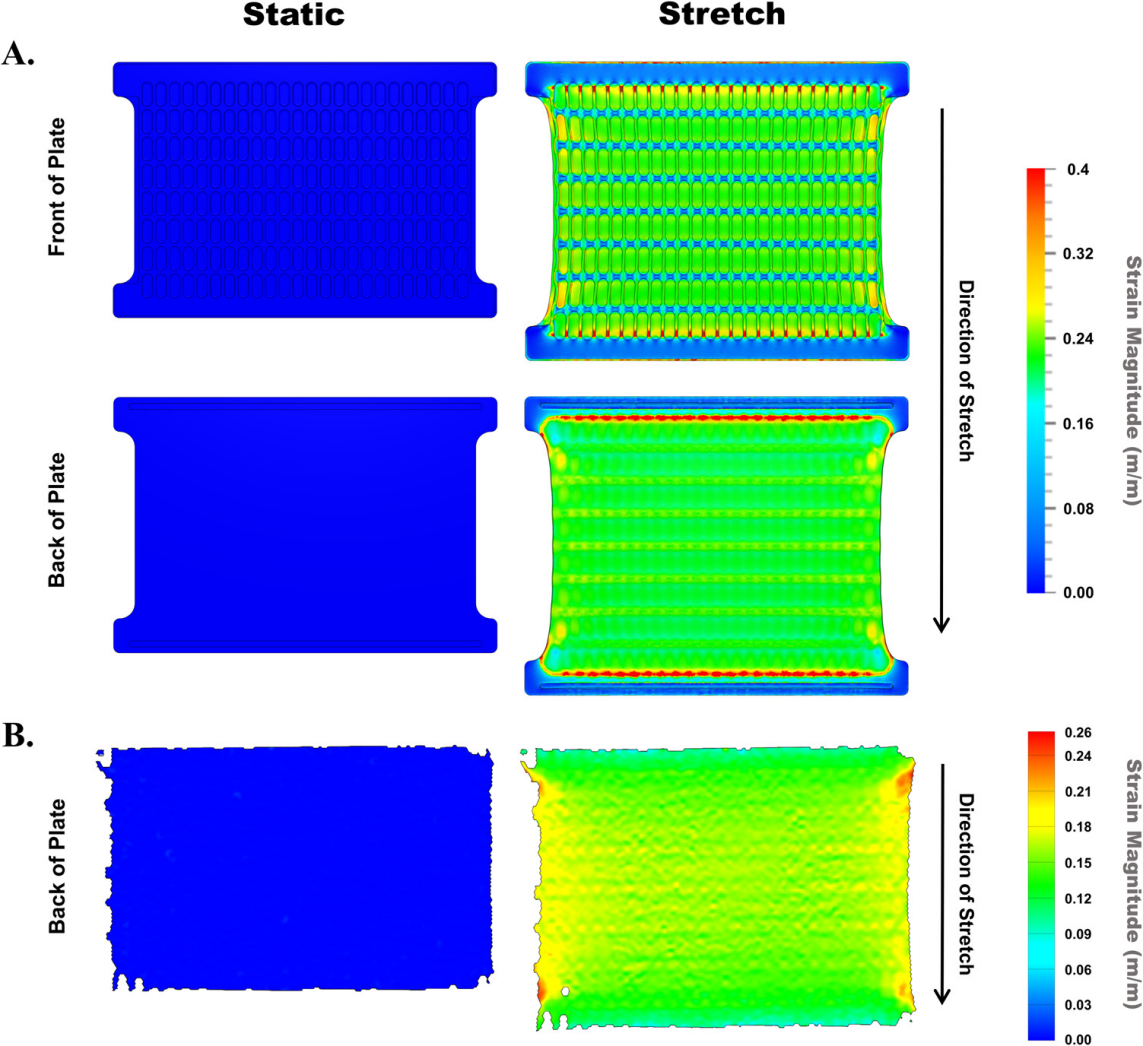
Finite element modeling and digital image correlation for intra-plate strain distribution. (a) Finite element analysis of the 192-well stretch plate under a static 20% displacement along the plate column axis (direction of stretch) was created in SimScale CAE software to model the commanded 20% linear stretch applied by the machine and to predict intra-plate strain. The simulated maximum strain (principal strain 3) was computed and plotted using a strain magnitude color scale of 0–0.4 m/m. A pre-displacement baseline strain plot (static) was included to compare static vs stretch conditions. Simulations were performed for both the front and back sides of the plate and respective plots shown. (b) The intra-plate displacement field was experimentally tested on the back side of the stretch plate. Major strain was computed for stretch plates subjected to 20% cyclic stretch at 1 Hz, using images captured by the Aramis camera system and analyzed with Inspect Corelate commercial digital image correlation (DIC) software. Computed maximum strain was plotted using a strain magnitude color scale of 0–0.26 m/m, for pre-stretch maximum strain and for the peak of commanded 20% stretch applied by the stretch machine, to compare static vs stretch conditions.

Application of high throughput stretch to cells *in vitro* was investigated next using the stretch device and plate. Though the stretch plate has a high percent stretch capacity (supplementary material Fig. 1), application of 20% stretch was chosen as it is within the range of strain considered physiologic for chondrocytes.^91,92^ To first determine intra-plate strain during applied stretch to the plate, finite element modeling (FEM) of plates under a static 20% displacement was performed using prediction software on both the front side and back side of the plate [Fig. 3(a)]. The maximum strain on the well top surface, where cells attach and maintain contact, was predicted to be uniform for wells across the majority of the plate (top = 0.20–0.23 m/m and bottom = 0.17–0.19 m/m). There were high strain values predicted for the well surfaces from the first and last row of the plate; however, these are likely to arise from the proximity to the aluminum clamps included in the simulation, which constrain the silicone material at these contact points (predicted maximum strain top = 0.23–0.26 m/m and bottom = 0.18–0.30 m/m). In addition, the outer two columns showed higher maximum strain values and greater variability (top = 0.23–0.30 m/m and bottom = 0.19–0.26 m/m), due to increased physical deformation transverse to the stretch direction compared to other regions. Next, strain was experimentally tested using digital image correlation (DIC) [Fig. 3(b)]. Due to the inability of the cameras and DIC to capture intra-plate strain inside complete wells across the full plate at once, the back side of the plate was used for analysis. The back side of the stretch plate was patterned with aerosol spray black paint to create a stochastic speckling pattern for tracking points in motion and computing strain (supplementary material Video 2). DIC was performed on three plate replicates stretched to 20% at 1 Hz where images were simultaneously collected at a frequency of 50 Hz for 5 s to capture multiple cycles of stretch (supplementary material Video 2), and average intra-plate maximum strain computed for each replicate (Table I). The DIC average strain data (Table I) and strain field patterns (not shown) show high repeatability across replicates. The DIC intra-plate strain distribution pattern [Fig. 3(b)] is consistent with the FEM simulation [Fig. 3(a)], showing strain variation across the plate, specifically in the outer columns. Some differences in the average strain were identified between the FEM and DIC, with DIC showing a lower average strain than the model simulation.

**TABLE I.** Digital image correlation values. Digital image correlation (DIC) was performed on three technical replicate plates subjected to 20% cyclic linear stretch at 1 Hz. Computed DIC maximum and minimum average strain and standard deviation values for each replicate plate are listed in the table.

Digital image correlation values	Plate 1	Plate 2	Plate 3
Maximum strain average	16.058	15.138	15.552
Maximum strain standard deviation	2.462	2.147	2.238
Minimum strain average	−2.351	−2.281	−2.407
Minimum strain standard deviation	1.83	1.674	1.904

**FIG. 4.**
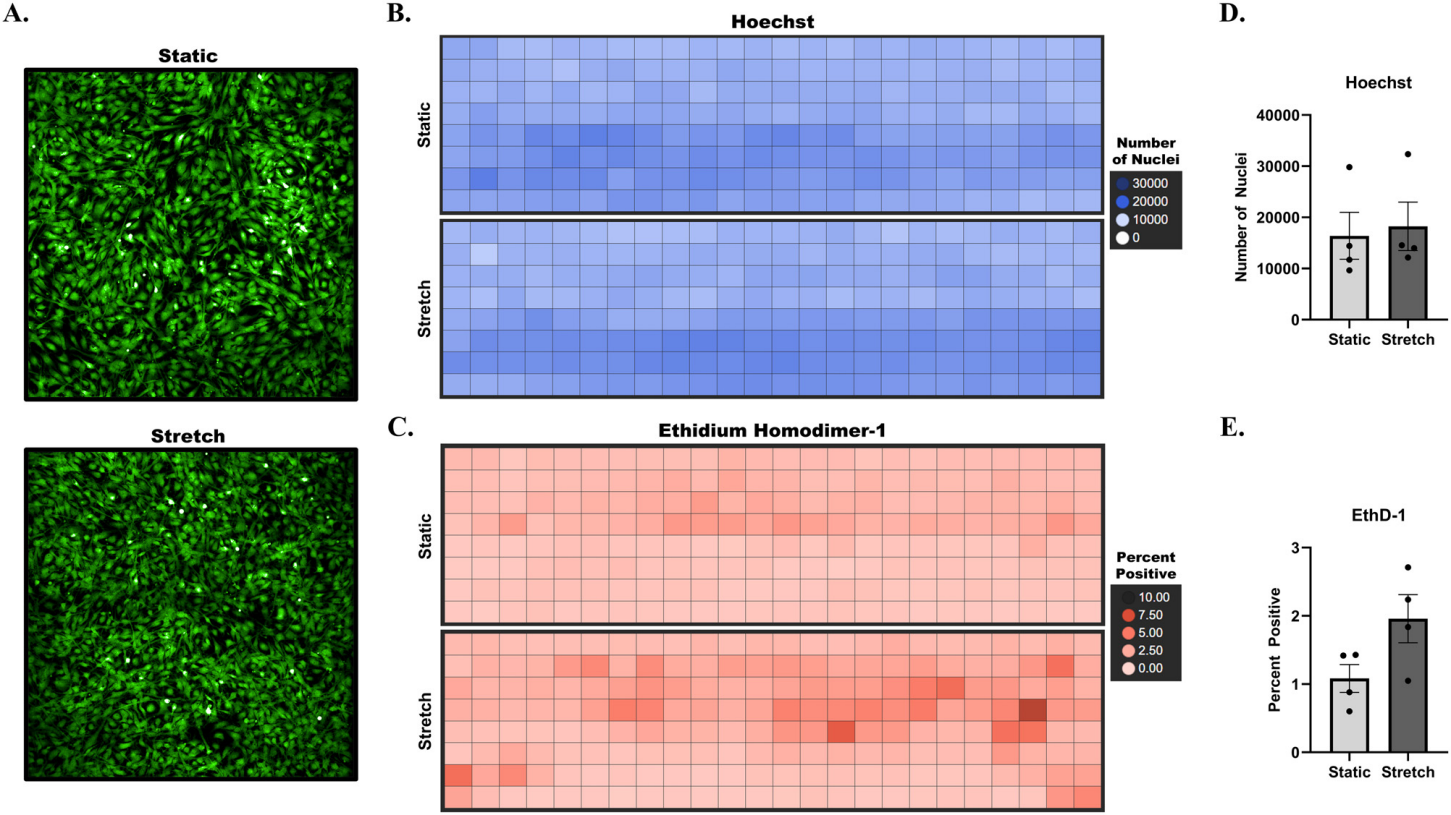
Live cell image analysis of EthD-1 with stretch. Human primary chondrocytes were subjected to 20% cyclic linear stretch at 1 Hz for 2 h and then co-stained with the nuclei stain Hoechst and the cell death and damage marker ethidium homodimer-1 (EthD-1). Full plates were scanned and imaged using the Opera Phenix High-Content Screening System for image stitching and z-stacking to capture the total cell population. (a) Representative images of cells stained with Calcein-AM from static and stretched plates are shown. (b) Total cell number in each of 192-wells across the static and stretch plates was visualized using cells positively stained with Hoechst and plotted in plate view from a representative plate using TIBCO Spotfire software. A blue-shaded gradient color scale representing the total cell number from 0 to 30 000 is used. (c) Percent cells positively stained with EthD-1 were plotted in plate view from a representative plate to visualize percentage of damaged cells per well across the stretch plate under static and stretch conditions. A red-shaded gradient color scale representing percent positive cells from 0 to 10 is used. (d) The total number of nuclei was counted, and plate average and standard error of the mean were plotted for four static and four stretch plates. (e) Percent positive cells were quantified, and plate average and standard error of the mean were plotted for four static and four stretch plates. Significance was performed in GraphPad Prism 9, using an unpaired t-test with Welch correction and Holm-Šídák method and p-value < 0.05. No significant differences were determined for (c). (Hoechst p-value = 0.787) or D. (EthD-1 p-value = 0.086).

To test whether application of dynamic stretch was damaging to cells, human primary chondrocytes were subjected to 20% cyclic stretch for 2 h, stained with the cell death and damage marker ethidium homodimer-1 (EthD-1), and live-cell imaged using a high content system. EthD-1 is a cell impermeant dye added to cell media that is weakly fluorescent. After crossing compromised plasma membranes of damaged or dead cells, it can bind to DNA with high affinity and emit fluorescence. Co-staining with Hoechst for labeling of nuclei was used to determine the total number of cells per well. No obvious gross cell detachment or other morphological changes were noted under initial observation of cell images [Fig. 4(a)]. Representative plate view plots were then used to visualize the total number of cells [Fig. 4(b)] and percent positive EthD-1 [Fig. 4(c)] per well across the plate for static and stretch conditions. Some trends were noted for EthD-1 with stretch; however, the effect was minor in a subset of wells. Importantly, experimental replicates for static and stretch were quantified and plotted, and showed no significant differences for the total number of cells [Fig. 4(d), p-value = 0.787] or for EthD-1 percent positive cells [Fig. 4(e), p-value = 0.086] between static vs stretch. Based on these data, 20% cyclic stretch for 2 h does not result in gross cell detachment or induce significant cellular damage.

**FIG. 5.**
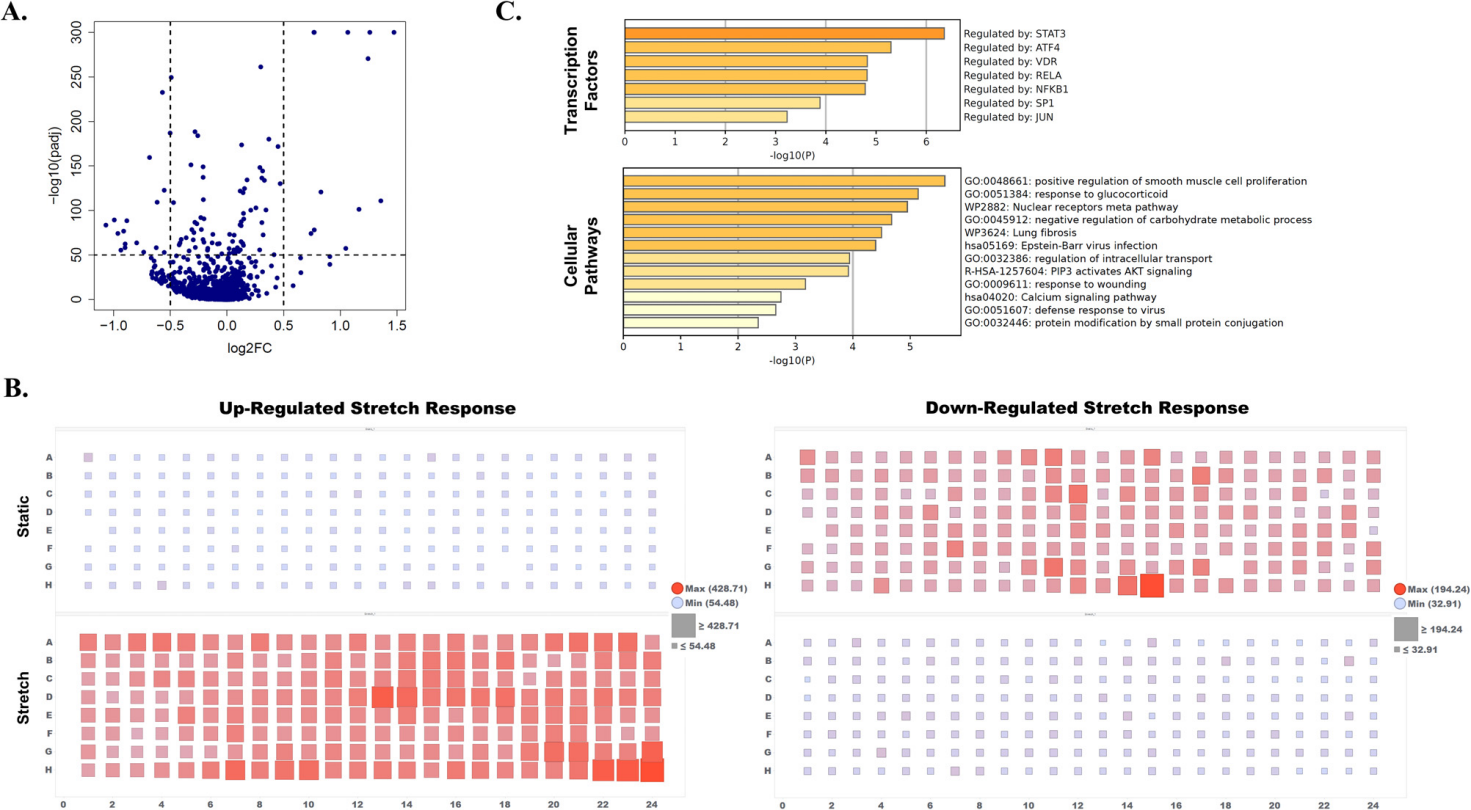
Stretch-stimulated gene signature. RASL sequencing of cellular landmark and chondrocyte genes was performed to identify stretch responsive genes. (a) Volcano plot shows differentially expressed transcripts with stretch compared to static conditions, from human primary chondrocytes subjected to 20% cyclic linear stretch at 1 Hz for 2 h. Stretch genes were defined as above thresholds set to −log10padj > 50 and |log2FC| > 0.5. (b) Gene expression plotted in plate view using TIBCO Spotfire software is shown as average reads per million (RPM) for each well across the static and stretch plates, for either the up-regulated gene set (up-regulated stretch response, left) or the down-regulated gene set (down-regulated stretch response, right). Average RPM values are visualized using color and shape, scaling shown in respective graph legends. (c) Cell pathway and transcription factor enrichment based on the identified stretch responsive genes were determined using Metascape (metascape.org) online software.

To explore biological readouts for the stretch device, stretch-induced gene expression was probed to build upon prior work in chondrocytes.^80–90^ Human primary chondrocytes were subjected to 20% cyclic stretch for 2 h and immediately after stretch cell lysates were collected from the stretch plates and static control plates using the Agilent Bravo Liquid Handling Platform for subsequent RNA extraction and sequencing analysis. Broad characterization of differential gene expression with stretch was performed by measuring landmark cellular transcripts, previously developed as a reduced representation of the transcriptome, termed L1000.^93^ Chondrocyte anabolic and catabolic genes were also included for cell type specificity.^51,94–98^ Transcriptional expression per well was measured by RNA-mediated oligonucleotide Annealing, Selection, and Ligation with Next-Gen sequencing (RASL-seq) technology.^99,100^ This RASL-seq design for general capture of large number of signal transduction pathways combined with cell-type genes, provides a more cost-effective analysis than other sequencing methods (e.g., full RNA-seq). Differentially expressed transcripts from stretch vs static conditions were plotted, and thresholds set to −log10padj > 50 and |log2FC| > 0.5 to determine stretch responsive genes [Fig. 5(a), Table II). There were 29 stretch responsive genes identified (12 up-regulated and 17 down-regulated). The stretch gene response was visualized in plate view to compare well-by-well gene expression under static vs stretch conditions [Fig. 5(b)]. Gene expression was plotted as average reads per million (RPM) for each respective up-regulated gene set or the down-regulated gene set [Fig. 5(b)]. While the plate view reveals some intraplate expression differences for any given plate (e.g., static or stretch in up-regulated or down-regulated graphs), the stretch-stimulated average gene expression shows clear separation from the static well-counterparts across the plate. Next, *in silico* analysis was performed for the gene set using Metascape^101^ to determine pathway and transcription factor enrichment based on the stretch responsive genes (combined up-regulated and down-regulated transcripts). Significantly enriched transcription factors and cellular pathways were plotted [Fig. 5(c)]. Of note, STAT3, RELA, and NFKB1 transcription factors identified are considered players in key pathogenic signaling pathways in osteoarthritis.^102^ The reported stretch gene signature is an example demonstration of how the stretch device could be used in high throughput screening against differential gene expression readouts with stretch in chondrocytes.

**TABLE II.** Stretch responsive genes. RASL sequencing of cellular landmark and chondrocyte genes was performed for chondrocytes subjected 20% cyclic linear stretch at 1 Hz for 2 h. To identify stretch responsive genes, differentially expressed transcripts with stretch compared to static conditions were defined as above thresholds set to −log10padj > 50 and |log2FC| > 0.5. There were 29 stretch responsive genes identified (12 up-regulated and 17 down-regulated).

Gene	log2FC	n.log10padj
CCN2	1.47	300.0
FOSB	1.36	110.7
GADD45B	1.26	300.0
IER3	1.24	270.5
NFATC2	1.16	101.3
BHLHE40	1.07	300.0
IL6	1.05	57.4
HBEGF	0.83	120.7
GPRC5A	0.77	78.1
SKIL	0.77	300.0
HES1	0.77	300.0
PTGS2	0.74	74.1
USP7	−0.50	186.9
PHKB	−0.55	52.8
CASC3	−0.55	122.7
G3BP1	−0.57	232.6
SENP6	−0.62	109.2
FGFR2	−0.62	58.1
KDELR2	−0.68	159.5
UBE3B	−0.73	53.2
TCTN1	−0.80	63.6
NET1	−0.88	88.4
UFM1	−0.90	62.4
PROS1	−0.90	58.2
LBR	−0.91	76.7
DDIT4	−0.94	55.4
USP6NL	−0.96	74.2
SYPL1	−0.99	89.3
XPO7	−1.07	83.5

## DISCUSSION

Here, we report the fabrication of a stretch device and 192-well linear stretch plate for applications in mechanobiology research. The development of a high throughput device that models stretch is relevant to a variety of organs, such as heart,^67,68^ blood vessels,^66^ airway,^64,65^ bladder,^24^ colon,^25^ skeletal muscle,^63^ tendon,^55,62^ and cartilage.^60,61^ The use of a stretch system for *in vitro* models is therefore not limited to one cell type. The stretch plate, with ANSI standard plate contour dimensions, is automation compatible for easy integration into screening systems to facilitate high throughput endpoints such as target ID and drug discovery. While other stretch devices have been developed previously for high throughput and screening,^103–107^ the current 192w stretch plate is of higher well capacity than other reported 96-well format systems and provides this screening advantage. Moreover, the stretch plate silicone elastomer resin design could facilitate large scale injection-molding manufacturing for rapid production and bolster high throughput research. The chondrocyte stretch gene signature, characterized by L1000 gene expression profiling and RASL-seq, and with adequate intra-plate consistency relative to static gene expression, illustrates feasibility of utilizing transcriptional readouts with this system. Alternative stretch readouts, such as protein expression,^103,107^ cell proliferation,^17,108^ morphological alignment with stretch,^104,106,109^ or calcium signaling,^110^ may also be employed. Overall, the stretch device and 192-well linear stretch plate have broad applicability for mechanobiology-based research.

The proof-of-concept chondrocyte stretch-induced gene set includes 29 genes (12 up-regulated and 17 down-regulated), several of which have known functions or associations in cartilage biology and osteoarthritis (OA). CCN2 was the top differentially expressed stretch gene. Previously reported to be up-regulated by stretch in chondrocytes,^87,111^ CCN2 is a target gene^58^ of the stretch sensitive^108^ transcriptional co-factor YAP. Interestingly, the absence of YAP can promote cartilage degradation.^112^ CCN2 was shown to have high expression in moderate to severe OA cartilage and in proliferating chondrocytes^113^ and was reported to be a driver of ECM production.^114^ Some evidence supports a role for the stretch gene HES1 in facilitating OA development, as deletion of HES1 conferred resistance to OA *in vivo.*^115^ Interestingly, HES1 was reported to target expression of IL6,^115^ another stretch responsive gene in the gene signature. Like HES1, IL6 has been shown to promote OA and cartilage degradation *in vivo* with an additional role in mediating OA pain.^116^ Other OA-related stretch genes of interest include BHLHE40, which may have a role in the OA onset,^117^ and XPO7, identified as a driver of cellular senescence^118^ that has implications for OA.^119^ In contrast to OA-facilitating genes, several genes in the stretch signature may provide protection against OA. The calcium activated transcription factor NFATC2^120^ is another high-fold up-regulated stretch gene. The lack of NFATC2 engenders spontaneous and early-onset OA in mice.^121^ Similarly, HBEGF provides resistance to the development of OA via chondroprogenitor expansion.^122^ However, GADD45B shows low expression in OA patients and may be necessary for COL2 production and cell survival.^123^ The differential expression of these chondrocyte stretch responsive genes fits well into what is known about the essential role of mechanical signaling in balancing tissue homeostasis vs development of osteoarthritis.^49–52^ Moreover, several transcription factors upstream of the stretch gene signature, identified by *in silico* analysis here (STAT3, RELA, and NFKB1), mediate osteoarthritis signaling pathways.^102^ However, what specific roles cell stretch or this curated stretch gene signature play in the promotion or health or disease have yet to be established and require further research.

Our FEM simulations and DIC findings reveal variations in strain field across the plate, specifically near the outer columns of the plate. Moreover, average strain from DIC analysis showed a lower average strain than that predicted by the FEM simulation. Possible explanations for differences in strain values include potential error in DIC methods, or plate slippage occurring from the clamp that is not captured by the model. Adjusting command percent stretch could correct for this in future experiments. Interestingly, the intra-plate strain variation edge effects do not seem to have an effect on stretch-dependent gene expression in chondrocytes in perimeter wells from the current study, as we do not see a similar intra-plate pattern for gene expression [Fig. 5(b)]. However, users may want to consider these variations for other cell types and phenotypic readouts.

As with any *in vitro* mechanobiology device, the application of stretch to cultured cells and their substrates presents a simplified model of the mechanical strains that occur throughout multiple dimensions in whole tissue *in vivo*. Indeed, there has been an emphasis in research for the development of three-dimensional (3D) multi-cellular organoids for better physiologic representation *in vitro*, including the importance of 3D mechanical signals of the ECM such as confinement, viscosity, and stiffness.^124^ Cell type stiffness specificity is almost entirely ignored in *in vitro* cell culture, as typical culture plastic stiffness (∼1000 MPa) is far beyond the ranges of most tissue types *in vivo*,^125^ which can have long term cellular consequences for function and identity.^19,31,41,42^ In contrast, our stretch plates with elastic modulus of 1.3 MPa are much closer in range to that of human cartilage (1.03 ± 0.48 MPa).^126^ Still, many other aspects of cellular organization and mechanical signaling in 3D are not recapitulated in monolayer culture, and for this, the stretch device would need modification. It is feasible that future iterations of the stretch device could incorporate single cells or cell organoids suspended in 3D matrix or hydrogels and apply tensile stretch. The depth of the wells could certainly accommodate this, with some cost-efficiency achieved via small well volumes. Such a system could not only include physiologic multi-cellular interaction, stiffness, confinement, and viscosity, but also apply complex multi-axial mechanical strains.

Stretch of chondrocytes in 2D aims to model the pericellular ECM deformations,^127^ tensile strains,^60,61^ and cell shape changes^127–133^ that occur during 3D compression of cartilage matrix and embedded cells. While there are limitations and assumptions, 2D stretch has been employed to investigate chondrocyte mechanobiology. Importantly, differential expression of anabolic and catabolic genes in response to chondrocyte stretch^80–90^ is desired and relevant phenotypes for a cartilage mechanobiology model system and bolster acceptance of 2D stretch for research. Of note, the genes included in the current study's stretch gene signature in response to 2 h of 20% stretch, do not include many of the previously reported chondrocyte stretch genes. However, this is not surprising as chondrocyte mechanoresponsiveness is time-dependent, magnitude-dependent, and culture condition-dependent,^87–90,134,135^ and users can adjust these variables for desired outcomes. The stretch device and gene signature developed here under specific stretch conditions seem to be in part an osteoarthritis-relevant cellular response. For any *in vitro* model, however, including gene signatures and other stretch phenotype readouts, robust iterative *ex vivo* and *in vivo* validation is required to establish physiologic or disease relevance. From a mechanobiology perspective, there are a wide array of tools developed at the *in vivo*, *ex vivo*, or single cell level that can be employed during the validation process, each of which provides advantages and disadvantages. For cartilage chondrocytes, this includes rodent treadmill running^136–139^ and joint loading;^140–146^ tissue explant or 3D cell-seeded construct compression;^75–77,130,133^ and single cell compression,^147^ membrane stretch,^148,149^ shear,^150^ or substrate deflection.^134^ Adding to this mechanobiology toolbox, the stretch device and 192-well linear stretch plate described here can provide additional throughput at the screening stage.

## CONCLUSIONS

A high throughput stretch device and silicone stretch plate were developed for applying linear stretch to cultured cells *in vitro*. Integration of the 192-well stretch plate with commonly used automated platforms was successfully performed, demonstrating compatibility with automation for high throughput endpoints such as target ID and drug discovery. While multiple cell types and phenotypic readouts have been previously established for stretch, a mechano-transcriptional stretch response was tested here as a proof-of-concept readout for the 192-well stretch plate. Using chondrocytes as a representative mechanoresponsive cell type, the gene signature identified contains stretch responsive genes that are relevant to cartilage homeostasis and disease. This stretch system provides a throughput advantage and broad applicability in mechanobiology-based research.

## METHODS

### Primary chondrocyte isolation

Fresh dissected human knee articular cartilage was acquired from a healthy 34 year old male donor with no medical history of arthritis or joint disease, within 48-72hrs postmortem, curated by Lonza. Harvested cartilage was cut into small pieces and then digested in 40 ml of 0.2 *μ*m filtered (Thermo Fisher Scientific, 564-0020) DMEM/F-12 (1:1) +L-Glutamine/+HEPES (Gibco, 11330–032) and 1% Antibiotic-Antimycotic (100× Gibco, 15240–062, Penicillin/Streptomycin/Amphotericin B), containing 0.1% Pronase Protease (Millipore, 53702-25KU) for 1 h at 37 °C, without agitation. Cartilage pieces were then centrifuged, pronase DMEM/F-12 solution removed, and were resuspended in 0.4% Collagenase II (Worthington, LS004177 5G) DMEM/F-12 solution overnight at 37 °C, without agitation. The following day cells were filtered using a 100 *μ*m cell strainer (Corning, 431752) and washed three times using 650 g × 5 min centrifugation and 1X PBS (Gibco, 20012-027) with 2% Antibiotic-Antimycotic (100X Gibco, 15240-062). Cells were then resuspended in Chondrocyte Growth Medium (Lonza Kit CC3216: CC3217 + CC4409) for cell culture. On average, 6.04 × 10^6^ human chondrocytes per gram tissue were acquired.

### High throughput stretch device and stretchable labware fabrication

Stretch devices consisting of a linear drive with one fixed and one moving plate clamp were designed and manufactured in house. Clamps were designed to grip the first and last row of each plate as well as an interlocking feature on the plate's underside and two flanges on either end. Clamping force was applied by tightening two screws per clamp to a pre-set hard stop to avoid crushing the plate. Devices were operated through a LabVIEW user interface (National Instruments, LabVIEW 2016), and motion signals were communicated to the motor drive through a USB DAQ analog output signal (National Instruments, USB-6003). Devices were placed inside an incubator for the duration of all stretch protocols, and stretch percentages were calculated as a ratio of maximum clamp spacing to the initial clamp spacing. Stretch was applied as sinusoidal waveform oscillating at 1 Hz offset such that the peak-to-peak amplitude represented the total stretch percentage over baseline length. *In vitro* stretch variables of 20% cyclic linear stretch at 1 Hz for 2 h were used here and are similar to those reported previously.^81,151^

Stretchable 192 well assay plates were injection molded (BOY100E) using NUSIL MED-4930 silicone elastomer resin mixed at a ratio of 1:1 part A to part B (Graco Fluid Automation F4-5) in a custom steel mold. Plates were plasma cleaned with O_2_ gas for 3 min (Nordson March, AP-300) and immediately placed in a vacuum chamber to co-incubate with vaporized (3-Aminopropyl)triethoxysilane (Millipore Sigma, 440140) for 2 h. Following functionalization, plates were prepared for chondrocyte adhesion by a 30 min room temperature incubation with 2% aqueous glutaraldehyde solution (Millipore Sigma, 340855), followed by several washes in DI H_2_O and, finally, a 12 h incubation with 0.15 mg/ml type 1 rat tail collagen (Advanced Biomatrix, RatCol-5153) at 37 °C. Plates were then washed, placed directly under a high intensity UV-C lamp for 15 min to sterilize (Lind Equipment, LE6725UVC-1P1C), and stored at 4 °C until use. Plates were designed in 192-well format (8 rows × 24 columns), one half the capacity of a 384-well plate, where each well in a stretch plate corresponds to two wells in a 384-well plate. The external contour of the stretch plate fits ANSI standard plate dimensions. Individual well surface area is 16.92 mm^2^, and well bottoms have high optical clarity (ability to capture fluorescent images up to 20×). An aluminum plate holder was constructed to support the plate for use in automation devices such as GNF Systems Plate Washer-Dispenser, Beckman Coulter ECHO Acoustic Liquid Handlers, Agilent Bravo Liquid Handling Platform, and Opera Phenix High-Content Screening System.

### Imaging

For automated cell seeding density and cell damage imaging assays, cells were seeded at 75 000 cells per well using a GNF Systems Plate Washer-Dispenser and cultured overnight. To visualize cell monolayer density, a nuclear stain (Hoechst 33342, Thermo Fisher Scientific, 62249, 361 nm) was added to live cells at 1:1000 for 30 min and then imaged. To visualize cell damage under stretch vs static conditions, the cell death and damage marker ethidium homodimer-1 (Invitrogen, L3224; ethidium homodimer-1 or Eth-B, 514 nm) was added to live cells at 1:500 for 30 min and then imaged. EthD-1 is a cell impermeant dye added to cell media that is weakly fluorescent in unbound form. After crossing compromised plasma membranes of damaged or dead cells, it can bind to DNA with high affinity and emit fluorescence. Plates were imaged on a PerkinElmer Opera Phenix High-Content Screening System using a 10× objective. To image the entire plate, 12 sequential XY-plane images with 2% overlap were captured for stitching, and water sequential Z-plane images at 12.5 *μ*m distance (total of 50 *μ*m overall height) were captured for z-stacking. This was completed for both Hoechst and EthD-1, and for each of the 192 wells across the plate. To determine if stretching cells induced cell damage or affected cell viability, EthD-1 quantification was measured as percent positive cells per well, then an average of percent EthD-1 positive cells across the plate was determined for each of four static and four stretch repeats. Hoechst co-staining was used to quantify cell number, where Hoechst-stained cell nuclei were counted in total for each of the 192 wells across the plate and for each of four static and four stretch repeats. Significance was determined using an unpaired t-test with Welch correction and Holm-Šídák method and p-value < 0.05 threshold, performed in GraphPad Prism version 9.5.1. GraphPad Prism version 9.5.1 software was used for plotting plate averages and standard error of the mean (SEM) in graphs. TIBCO Spotfire Analyst 12.0.4 LTS HF-020 software was used for plotting plate view images of number of nuclei or percent positive cells per well across a representative plate.

### Finite element modeling and digital image correlation

Finite element modeling of the 192-well stretch plate was created in SimScale CAE software (SimScale GmbH) using identical plate dimensions. A second order finite mesh with 710 223 tetrahedral elements and 1 177 555 nodes was generated using an automatic mesh generator tool that resulted in an average aspect ratio of 1.61 (σ 0.58). The silicone plate material was considered to be incompressible, having a Young's modulus of E = 1.30 MPa, Poisson's ratio ν = 0.45, and density ρ – 1130 kg/m^3^. The stretch machine aluminum clamps that interface with the stretch plate in four contact areas (top and bottom of the plate along the first and last row axes) were included in the simulation using a hardware Young's modulus of E = 70 GPa, Poisson's ratio ν = 0.34, and density ρ – 2700 kg/m^3^. Fixed support boundary conditions were applied to hold the two stationary clamps in place and to instruct the model to only move in one direction. A static remote displacement condition was used to model the 20% linear stretch applied by the machine along the plate column axis. The simulated maximum strain (principal strain 3) was computed and plotted.^152^

The displacement field across the plate with stretch was experimentally tested using digital image correlation (DIC)^152,153^ (https://idics.org/guide/). DIC measurements of the stretch plates were performed using the Aramis camera system and analyzed with INSPECT Correlate commercial DIC software (ZEISS Quality Suite 2023 version, Trilion Quality Systems, King of Prussia, PA, USA). The back side of the stretch plates was first patterned with aerosol spray black paint to create a stochastic speckling pattern of fine points so the cameras and software can track points in motion in order to measure displacement and compute strain. The samples were backlit with two blue LEDs to reduce glare and create sufficient contrast of the speckle pattern on the translucent stretch plates. A reference photograph was taken at a pre-stretch timepoint, and a surface component was created from the pre-stretch reference photograph. Samples were then stretched to 20% at 1 Hz, while images were simultaneously collected at a frequency of 50 Hz for 5 s to capture multiple cycles of stretch. Three replicate plates were tested. Major (maximum) strain and minor (minimum) strains were computed across the plate to evaluate inter-plate strain variations with stretch.

### Sequencing and bioinformatic analysis

Immediately after 20% cyclic linear stretch at 1 Hz was applied for 2 h, two static and two stretch plates were lysed using a lysis buffer containing PK buffer (Thermo, 4489111) and Proteinase K Solution (New England Bio, P8107S) at a 24:1 ratio. Pooled barcoded PCR amplicons were quantified on a Qubit Fluorometer, and the correct size was confirmed on an Agilent TapeStation using a High Sensitivity D1000 assay. Samples were sequenced on a NextSeq 550 using 51 sequencing cycles to a read depth of approximately 300 000 aligned reads per sample well. Sequencing reads were demultiplexed using Illumina bcl2fastq software v2.20.0.422. Reads were aligned to a reference of expected ligation products with bwa version 0.7.17-r1188,^154^ and the numbers of reads assigned to each expected ligation product were counted with samtools version 1.11.^155^ Counts reads aligning to expected ligation products were normalized based on library size (CPM).

The per well sequencing approach utilized RNA-mediated oligonucleotide Annealing, Selection, and Ligation with Next-Gen sequencing (RASL-seq) technology.^99,100^ Selected genes for RASL-seq included chondrocyte specific anabolic and catabolic genes,^51,94–98^ as well as landmark transcripts developed as a reduced representation of the transcriptome sufficient to recover a majority of information in the full transcriptome (termed L1000).^93^ This RASL-seq design for general capture of large number of signal transduction pathways combined with cell-type genes, provides a more cost-effective analysis than other sequencing methods (e.g., full RNA-seq). Count matrix was obtained by mapping the raw reads to the probe sequences as previously described,^100^ and samples with <15 000 mapped reads were removed. Next, read counts were normalized by the total mapped reads, i.e., RPM (Reads Per Million mapped reads). Out of three probes designed for each gene, the one with the highest overall RPM was chosen as the measurement of that gene. The consistency of measurement was evaluated by inter-replicate similarity determined by the Pearson correlation coefficient of log2(RPM + 1) of all the genes. Samples with maximum inter-replicate similarity < 0.8, or >40% undetected probes compared to the best replicate, were considered as outliers, and excluded from downstream analysis. Batch effect across plates was removed using pyCombat,^156^ and then, quantile normalization was applied to adjust log2(RPM + 1) across replicates. Differential expression analysis was performed using limma^157^ in R (R Core Team (2021) A language and environment for statistical computing, R Foundation for Statistical Computing, Vienna, Austria, https://www.R-project.org/); stretch condition, stretch plate, and stretch plate location were used as covariates in the model to remove any bias from these variables. R language was used to display volcano plots. Final differentially expressed transcripts from stretch vs static conditions were determined using thresholds set to −log10padj > 50 and |log2FC| > 0.5 (Table II). Pathway and transcription factor enrichment analyses, and graph plotting, were performed by using Metascape (www.metascape.org),^101^ where −log10padj > 50 and |log2FC| > 0.5 differentially expressed stretch genes were background corrected against our targeted RASL-seq gene set and analyzed. TIBCO Spotfire Analyst 12.0.4 LTS HF-020 software was used for plotting plate view images of average RPM for each well across the static and stretch plates, for either the up-regulated gene set or the down-regulated gene set (Table II).

## SUPPLEMENTARY MATERIAL

See the supplementary material for additional images and video of the device applying stretch to the 192-well stretch plate, for images of the plate clamp, for a video containing additional digital image correlation analysis material, and for a three-Dimensional Portable Document Format (3D-PDF) of the stretch device, stretch plate, and plate holder designs.

Supplementary material video 1: linear stretch plate video: the video shows the 192w silicone stretch plate clamped in both the static fixed clamp (left side of the plate) and moving arm with clamp (right side of the plate) of the machine. The plate is being subjected to 20% cyclic linear stretch at 1 Hz.

Supplementary material video 2: real-time digital image correlation video: the video shows the stretch plate subjected to 20% cyclic linear stretch at 1 Hz and temporal changes in intra-plate displacement field of strain computed by digital image correlation (DIC). The plate is patterned by aerosol spray black paint to create a stochastic speckling pattern of fine points for DIC analysis. The DIC computed strain is overlayed on the patterned stretch plate with a strain magnitude color scale of 0–0.26 m/m. The graph shows changes in average intra-plate strain through time.

Supplementary material Fig. 1: Stretch plate percent capacity: images show the 192-well linear stretch plate subjected to 20% stretch and 50% stretch, compared to static pre-stretch length. The maximum stretch capacity of the stretch plate is 50%.

Supplementary material Fig. 2: Stretch plate holder. Computer-aided design (CAD) images show the aluminum plate holder alone (a) and with the plate secured to the holder (b). The plate holder was constructed to support the stretch plate and allow automation gripping mechanisms to firmly clasp the plate. The plate holder has two metal protrusions along the row axis that fit into the underside of the top and bottom of the silicone plate, securing the plate into place.

Supplementary material dataset 1: Three-Dimensional Portable Document Format (3D-PDF). The dataset includes a three-Dimensional Portable Document Format (3D-PDF) of the stretch device, stretch plate, and plate holder designs.

## Data Availability

The data that support the findings of this study are available within the article and its supplementary material.
